# Fungi as Endophytes in *Artemisia thuscula*: Juxtaposed Elements of Diversity and Phylogeny

**DOI:** 10.3390/jof4010017

**Published:** 2018-01-27

**Authors:** Andreea Cosoveanu, Samuel Rodriguez Sabina, Raimundo Cabrera

**Affiliations:** Department Botanica, Ecologia & Fisiologia Vegetal, Universidad de La Laguna, 38206 La Laguna, Tenerife, Spain; samuelrguezsabina@gmail.com (S.R.S.); rcabrera@ull.edu.es (R.C.)

**Keywords:** *Artemisia*, fungal endophytes, biodiversity, phylogeny

## Abstract

*Artemisia* is a plant genus highly studied for its medicinal applications. The studies on the associated fungal endophytes are scarce. Ten plants specimens of *Artemisia thuscula* from Tenerife and La Palma were sampled to isolate the endophytic fungi. Identification of the endophytic fungi was based on morphology, Internal Transcribed Spacer (ITS) and Large Subunit (LSU) regions sequencing and indicates 37 fungal species affiliated to 25 fungal genera. Colonization rate varied among plants (CR = 25% to 92.11%). The most dominant colonizers found were *Alternaria alternata* (CF = 18.71%), *Neofusicoccum* sp. (CF = 8.39%) and *Preussia* sp. (CF = 3.23). Tendency for host specificity of most endophytic fungal species was observed. Sorensen–Dice index revealed that of 45 cases in the matrix, 27 of them were of zero similarity. Further, only one case was found to have 57% similarity (TF2 and TF7) and one case with 50% similarity (TF1 and TF4). The rest of the cases had values ranging between 11% and 40% similarity. Diversity indices like Brillouin, Margalef species richness, Simpson index of diversity and Fisher’s alpha, revealed plants from La Palma with higher values than plants from Tenerife. Three nutrient media (i.e., potato dextrose agar―PDA, lignocellulose agar―LCA, and tomato juice agar―V8) were used in a case study and revealed no differences in terms of colonization rate when data was averaged. Colonization frequency showed several species with preference for nutrient medium (63% of the species were isolated from only one nutrient medium). For the phylogenetic reconstruction using the Bayesian method, 54 endophytic fungal ITS sequences and associated GenBank sequences were analyzed. Ten orders (Diaporthales, Dothideales, Botryosphaeriales, Hypocreales, Trichosphaeriales, Amphisphaeriales, Xylariales, Capnodiales, Pleosporales and Eurotiales) were recognized. Several arrangements of genera draw the attention, like *Aureobasidium* (Dothideales) and *Aplosporella* (Botryosphaeriales) which are clustered with a recent ancestor (BS = 0.97).

## 1. Introduction

Vascular plants species [1,2], aquatic plants and algae [3,4], mosses and ferns [5,6] examined to date are found to be hosts for endophytic bacteria and fungi [7]. Endophytic microorganisms have been isolated from different parts of plant-like scale primordia, meristem and resin ducts [8,9], leaf segments with midrib and roots, stem, bark, leaf blade, petiole [10], buds [11], and seeds [12]. Successful endophytic colonization is dependent on many factors including plant tissue type, plant genotype, the microbial taxon and strain type, and biotic and abiotic environmental conditions. Fungal endophytes aid plants to withstand and tolerate unfavorable environmental conditions [13,14] and also promote plant growth [15,16]. These inhabitants can produce the same or similar secondary metabolites [17,18,19,20] as their host and play vital roles *in vivo* such as signaling, defense, and regulation of the symbiosis [21]. Mainly investigations are based on their use as biochemical tools and the end products are to be used in pharmaceutics, industry, and agriculture.

*Artemisia* is a plant highly evaluated for medicinal and biopesticide traits. A survey of the literature shows this plant genus to be in the hot spot among researchers with over 11,200 publications in Scopus library. Even though *Artemisia* is a large plant genus with species producing a variety of interesting and active compounds, its endophytic communities are under investigated. The identification of the fungal endophytes in *Artemisia* spp. is made mainly based on morphological characterization and molecular analysis using nuclear ribosomal DNA sequences, including both the internal transcribed spacers and the 5.8S gene region. To the best of our knowledge, there have been only four studies which investigate the phylogenetic analysis of the *Artemisia* spp. fungal endophytes [22,23,24,25]. In terms of diversity, the studies are also scarce but interesting facts are brought to light in terms of diversity and plant colonization. For instance, Yuan et al., 2011 [26] performed a comparative study related to infection frequency between cultivated plants and wild plants of *Artemisia annua*. The results revealed slightly higher infection frequency of the endophytic fungi in cultivated roots (20.9%) than in native roots (16.7%). Further, authors described that the naturally regenerated roots harbored richer fungal genotypes, which supports the hypothesis that wild plant species are predisposed to host rich and novel mycoflora [27]. It is worth mentioning that Qian et al., 2014 [27] reported the presence of *Rhodotorula* sp. and *Fusarium* sp. in *Artemisia argyi* for the first time. The endophytic fungi associated with *Artemisia nilagirica* were investigated and one strain of *Pythium intermedium* (Oomycota) and one strain of *Rhizopus oryzae* (Mucoromycota) were isolated among the majority clade of Ascomycota [28]. Huang et al., 2009 [24] classified 108 fungal isolates obtained from three medicinal plant species *Artemisia capillaris*, *Artemisia indica* and *Artemisia lactiflora* using morphological identification and among the three plant hosts, the highest endophytic colonization rate occurred in *Artemisia capillaris*, which exhibited highest fungal diversity. Five fungal isolates belonging to *Aureobasidium pullulans*, *Ephelis*, *Pestalotiopsis*, and Pleosporaceae, were only recovered from *Artemisia capillaris*. *Xylaria* species was reported to be dominant endophytic fungi in *Artemisia indica*. Seven *Artemisia* species were sampled in two locations (Qichun and Wuhan in China) and 21 fungal endophytic species belonging to: *Diaporthe*, *Colletotrichum*, *Nigrospora*, *Botryosphaeria*, *Aspergillus*, *Penicillium*, *Neofusicoccum*, *Cercospora*, *Rhizoctonia*, *Alternaria,* and *Curvularia* were found [23]. The highest incidences of colonization frequency per plant host revealed *Nigrospora sphaerica* in *Artemisia* sp., *Nigrospora oryzae* in *Artemisia argyi*, *Alternaria alternata* in *Artemisia subulata* and *Artemisia tangutica* and *Botryosphaeria dothidea* in *Artemisia lavandulifolia*. The authors report for the first time *Nigrospora*, *Neofusicoccum* and *Curvularia* species in *Artemisia* spp.

*Artemisia thuscula* is an endemic plant of Canary Islands and community of endophytes housed inside its plant tissues remains unexplored. With the idea of exploring endemic medicinal plants for useful and underexplored fungal endophytes, we strategically pinned down to *Artemisia thuscula* that has been harboring in western areas of islands i.e., Tenerife and La Palma, for ages. Elements of phylogeny and diversity were framed for the strains obtained from both islands with a case study of Tenerife where diversity was intended to be enhanced by using different nutrient media and stem ages. Questions on host specificity were explored, having one plant species and various collection locations.

## 2. Materials and Methods

### 2.1. Plants Sampling

Plants of *Artemisia thuscula* species were collected from Canary Islands (La Palma and Tenerife). 10 plants specimens were sampled in total. Three plants were sampled from La Palma and seven plants were sampled from Tenerife; GPS coordinates are mentioned in Table 1. In situ, plants were observed for their healthy appearance prior to the sampling, only those individuals that did not show symptoms of attack by pest or disease were selected. From each plant only stems segments were cut, labeled and kept in paper bags inside zip-locked bags at T = 4–5 °C until transported to the laboratory and then processed within 24 h. Identification of the plant species was performed using classical morphological examination. The plants were deposited at the University of La Laguna (ULL) herbarium (TFC).

### 2.2. Fungal Endophyte Isolation

Surface sterilization method was used to suppress epiphytic microorganisms from the plant [23]. Thus, stem fragments were first washed with sterile water, then immersed in 70% ethanol for 1 min, followed by an immersion in 15% sodium hypochlorite for 1 min, again in 70% ethanol for 1 min and lastly were washed with sterile distilled water. To assure a successful sterilization, fragments were rolled on potato dextrose agar (PDA) medium and drops of last step sterilization water were poured on medium, as a control check for complete sterilization. After this process, plant material was dried on sterile blotting sheet, excised in pieces of 2 cm and cut longitudinally with a sterile scalpel. Segments were placed in PDA (Sigma-Aldrich, St. Louis, MI, USA) Petri plates amended with tetracycline (10 mg L^−1^). Plates incubated with the plant segments were incubated at 25 °C in the dark for two weeks and observed daily for fungal growth. When fungal outgrowth from the plant tissues occurred observations on emerged fungi were made. Only the fungi with different morphological characteristics were subcultured. Eventually, when an endophyte was acquired in pure culture it was preserved in Czapek medium (Fluka Analytical, Sigma-Aldrich), T = 5 °C and in glycerol (≥99.5, Sigma-Aldrich) 20% in deionized H_2_O, T = −32 °C and identified. To analyze the fungal diversity, each replicate of the distinct stem fragments was noted. To enhance bioprospection and diversity, variable nutritive media were utilized to incubate stem fragments (with ages less than one year and more than one year) of eight plants from Tenerife. Therefore, V8 tomato juice agar and lignocellulose agar (LCA) [29] media were additionally used. All the reagents were purchased from Sigma-Aldrich, except Agar Agar—GUINAMA (Valencia, Spain) and Potassium chloride—PanReac AppliChem (Barcelona, Spain).

### 2.3. Fungal Endophyte Collection and Maintenance

Every isolate and its plant origin were dully recorded for calculation of colonization rate from host, counting the same isolate identification only once if it emerges from the same plant segment. After purification of each isolate, it was subjected to microscopical observations followed by molecular analysis to identify at genus and/or species level. Isolates are presently maintained in three types of media: Czapek, T = 5 °C; mineral oil (Sigma-Aldrich), T = 5 °C and glycerol (Sigma-Aldrich) 20% diH_2_O, T = −80 °C. For short term use, fungal isolates were maintained on PDA, 25 °C.

### 2.4. Morphological Identification

Prior to taxonomic identification, a preliminary classification was made to avoid the selection of identical strains arising from the same plant individual, separating isolates into morphotypes. Observations targeted characteristics related to the colony and medium as: colony shape, texture and colour; exudates, medium colour and growth rate. For the microscopic observations, a strain was inoculated onto a PDA Petri plate and a sterile cover slide was attached at two centimeters. Once the growth of the fungus partially covered the cover slide, the slide was removed, inverted on a slide with cotton blue (for the slightly coloured colonies) and observed under microscope.

### 2.5. Molecular Identification

Out of several procedures for genomic DNA extraction, the most efficient protocol, although time consuming, was the one described by Shu et al., 2014 [20] to which the following modifications were made. Samples were centrifuged for 15 min at 12,000 rpm; after the chloroform (≥99.5, Sigma-Aldrich) procedure the upper phase was mixed with 10% Sodium acetate (ReagentPlus^®^, ≥99.0%, Sigma-Aldrich) and 60% Isopropyl alcohol (Aldrich ≥ 97.0%, Sigma-Aldrich), incubated for 10 min at −30 °C and centrifuged (10 min, 12,000 rpm). Finally, the pellet was washed twice with 75% ethanol (before maintained at −20 °C) and centrifuged (10 min, 12,000 rpm). The solvent was removed by evaporation, keeping the sample in the laminar flow cabinet. The purified DNA was suspended in 20 µL TE buffer (10 mM Tris-HCl, pH 8.0, 1 mM EDTA); all reagents were purchased from Sigma-Aldrich. RNase A was added, and the sample was incubated for 1 hour at room temperature (long-term storage at −32 °C).

The second protocol for DNA extraction involves no purification of DNA but acceptable results were garnered (around 50% samples succeeded). 20 µL of TE buffer was pipetted into a microtube and glass beads (diameter = 0.4–0.6 mm) were added to make up 3/4 of the reagent’s volume. A small quantity of fungal mycelium was added (2–5 mm/2–3 mg) with a needle. Samples were homogenized using FastPrep 24™ 5 G (MP Bio, Santa Ana, California, USA) at 4 m/s, 20 s. Subsequently samples were centrifuged at 13,000 rpm for 1 min and maintained on ice. One µL of the supernatant was used for the PCRs.

The third and fourth protocol involved two genomic DNA extraction kits. First one used was E.Z.N.A. Fungal DNA Kit according to the manufacturer indications (OMEGA bio-tek, Norcross, Georgia, USA) with overall good results (around 80% of the samples succeeded). The second one tested was Fungi/Yeast Genomic DNA Isolation Kit, according to the manufacturer indications (NORGEN Biotek, Thorold, ON, Canada) with overall good results also (approximately 70% of the samples succeeded).

The fourth protocol approaches nucleic acid extraction by application of silica coupled to magnetic particles, which is efficient and automated. Genomic fungal DNA was extracted using Maxwell 16 Mouse Tail DNA purification kit. The Promega kit is designed for automated DNA extraction from tissue samples using the Maxwell™ 16 platform (Promega BioSciences, San Luis Obispo, CA, USA). This protocol was performed at the University Institute of Tropical Diseases and Public Health of the Canary Islands, University of La Laguna.

Molecular identification of the fungal Dicarya strains was performed using ITS1 (5′-TCCGTAGGTGAACCTGCGG-3′) and ITS4 (5′-TCCTCCGCTTATTGATATGC-3′) primer pair to amplify the 5.8S rDNA and the two internal transcribed spacers ITS1 and ITS2 [30] for the majority of the samples and NL-1 (5′-GCA TAT CAA TAA GCG GAG GAA AAG-3′) and NL-4 (5′-GGT CCG TGT TTC AAG ACG G-3′) primer pair to amplify the 5′ end of 28S rDNA spanning domains D1 and D2) [31]. PCRs were performed in a total volume of 25 µL containing 10 ng genomic DNA, 0.5 µM primer, 200 µM dNTPs, 1X Buffer Taq, 0.0125U of Taq DNA Polymerase. For ITS sequences, PCR cycling parameters were carried out according to Shu et al. 2014 [20] with slight modifications: 94 °C for 2.5 min; 40 cycles of 94 °C for 30 s, 58 °C for 30 s, and 72 °C for 1 min; and a final extension at 72 °C for 10 min. For 28S rDNA domain, the PCR conditions were denaturation for 4 min at 95 °C followed by 45 s at 95 °C and then annealing for 45 s at 58 °C, 1 min at 72 °C, followed by an extension at 72 °C for 5 min. The final step was at 16 °C for 5 min. A total of 40 cycles were performed. All PCR products were detected by agarose gel electrophoresis (110V, 35 min, on 2% agarose gels, 1X TAE Buffer) loading 5 µL PCR product, 1 µL Loading Buffer (6X) and 2 µL SYBR Green I (Sigma-Aldrich; dilution 1:10,000). PCR and electrophoresis reagents were purchased from Sigma-Aldrich. PCR products were purified using GenElute™ PCR Clean-Up Kit (Sigma-Aldrich) and sequenced by Sequencing Services SEGAI (La Laguna, Spain). The sequences were run through the BLASTN search page using Megablast program (National Center for Biotechnology Information; Bethesda MD, USA) where the most identical hits and their accession numbers were obtained. Further, only ITS sequences were used for the phylogenetic analysis, therefore details on 28S sequenced strains are listed in Table 2.

### 2.6. Phylogenetic Analysis

ITS sequences [i.e., endophytic fungi—Table 3, their most similar hits from GenBank (NCBI, Bethesda MD, USA) and type sequences of the selected taxa] were aligned with the multiple alignment program ClustalW [32] as implemented in Mega 6.0 (Molecular Evolutionary Genetics Analysis) [33] and indels corrected manually to minimize alignment gaps [34]. Designated outgroup was *Caloscypha fulgens* (GenBank Accession No. DQ491483). After the exclusion of non-overlapping leading/trailing gaps the length of the alignment was 603 bps. Because of the high number of indels, these were recoded as a binary matrix by means of the simple indel coding algorithm [35], appending the fragments to the nucleotide data as additional characters, as implemented in FastGap 1.21 (Department of Biosciences, Aarhus University, Denmark) [36]. This “indel matrix” was used in all Bayesian and maximum likelihood analyses. Formerly, Gblocks program (hosted at www.phylogeny.fr) was used to eliminate poorly aligned positions and divergent regions [37]. Best-fit models were compared in jModelTest 2 according to Bayesian Information Criterion (BIC) [38]. Best fit according to the BIC criterion model (K80 + G) was selected to reconstruct the Bayesian tree. Bayesian Inference analysis was conducted with MrBayes 3.2.3 (hosted by Mobyle SNAP Workbench, North Carolina State University) [39] and run for 1 × 10^7^ generations with a sampling frequency of 100 generations. Of the resulting trees, the first 25,000 trees were discarded as burn-in and the following 75,001 were used to estimate topology and tree parameters. The percentage number of times a node occurred within these 75,001 was interpreted as the posterior probability of the node [40]. Convergence of the runs was indicated by an average standard deviation of split frequencies between duplicate runs of less than 0.01. The consensus trees were drawn using Treegraph 2 software (Institute for Evolution and Biodiversity, University of Munster, Germany) [41] and edited with Adobe Illustrator CS3 (Adobe Systems Incorporated, San Jose, CA, USA). 

### 2.7. Diversity Analysis

The colonization rate (CR%) was calculated as the total number of stem fragments in a sample (plant/nutritive medium) yielding at least one isolate divided by the total number of stem fragments in that sample. Colonization frequency (CF%) was calculated as the total number of fragments in a sample (plant/location) colonized by a species divided by the total number of fragments plated. For the diversity of endophytic fungi, the Margalef index, Brillouin index, Fisher’s alpha index and Simpson’s dominance index were used. Margalef index [42] measures species richness while Brillouin index combines richness and evenness. The Margalef index was calculated using formula d=(S−1)/ln N, where *S* is the number of species and *N* is the number of individuals in the sample. The Brillouin index [43,44] was calculated using formula: $\mathrm{HB}=\left(\ln N!-S\ln n_{i}!\right)/N$, where *N* is the total number of individuals, *S* is the number of taxa and *n_i_* is the number of individuals belonging to *i* species. Fisher’s logarithmic series model [45] is a species-abundant model and describes the relationship between the number of species and the number of individuals of those species. It was calculated using formula S=a×ln(1+n/a), where *S* is number of taxa, *n* is the number of individuals and a is the Fisher’s alpha. The dominance of Simpson [46] was calculated according to the formula $D=1-\sum \left[n_{i\left(n_{i}-1\right)}/N\left(N-1\right)\right]$, where *n_i_* is the number of individuals belonging to *i* species and *N* is the total number of individuals. The Sorensen–Dice coefficient of similarity [47,48] which expresses the beta diversity was employed to compare the similarity of endophytic fungi communities regarding species composition between two host plants, nutrient media and stem ages. The Sorensen–Dice coefficient is calculated with the formula QS=2C/(A+B) where *A* and *B* are the species numbers in samples A and B, respectively, and *C* is the number of species shared by the two samples. The Sorensen–Dice coefficient weighs more the joint occurrences than the mismatches and is expressed with values between 0 (no similarity) and 1 (absolute similarity). This index was used to assess host preference and spatial heterogeneity by describing the similarity of endophytic communities within ten host plants at distinct sampling sites. Nevertheless, as the coefficient analyses the presence/absence data, no judgments on abundance or rare taxa can be pursued. A binary matrix was produced and used to calculate the similarity matrix and to plot a dendrogram based on an unweighted paired group method of arithmetic average (UPGMA) cluster analysis. For the diversity indices, PAST software version 3.15 (copyright Hammer & Harper, Natural History Museum, University of Oslo, Norway) was used.

## 3. Results and Discussion

### 3.1. Fungal Endophytic Diversity in Artemisia Species

#### 3.1.1. Colonization Rate and Colonization Frequency of Endophytic Fungi in *Artemisia* *thuscula*

In this study, the employed analyses indicate that 37 fungal species and 25 fungal genera were isolated from 10 plants of *Artemisia thuscula*. Colonization rate (further CR) shows how much a plant can be colonized within predetermined conditions. It is valuable information as different plants showed distinct values of this index; therefore, low values could express plants poor in endophytic fungi culturable in the given conditions.

To calculate the colonization frequency (CF) of fungal endophytes in *Artemisia* species plants, we have considered same fungal endophytic species isolated from two or more plant fragments as being a distinct isolate belonging to the same species. Therefore, if the same species was isolated twice from the same plant fragment, it was considered only one time. This issue is to be expected at isolation moment, when no precise differentiation between the isolates can be defined, and only once purified and further analyzed then only the strain received a final identification. The CF% gives a hint over the distribution and abundance of a certain fungal species in a sample (i.e., plant/location/region). To know the “area” of the distribution and abundance of a certain endophytic fungal species, we have analyzed the data per plant individual or plant location, plant species, and plant region. Regions were grouped here as: La Palma Island and Tenerife Island. This way we can have an overview on where certain fungal species are more abundant or rare, as well as if there is a relation between their distribution and plant-specific parameters.

In *Artemisia thuscula*, only one plant out of 10 had a colonization rate value over 90% (LP2). The lowest values (CR% = 25) were recorded for three plants (TF8, TF7 and TF3). Interestingly, as per variable geographical location there is a considerable variation between La Palma Island and Tenerife Island, with the former having the most colonized plant individuals (Table 4).

*Artemisia thuscula* cannot escape of the “omnipresence” of *Alternaria alternata*, this species was isolated from eight plants but with relevant differences in the frequency, CF% = 15–50%. A notable presence is remarked here, *Neofusicoccum australe*, isolated from three plants at relatively high values (CF% = 25) when considering that the maximum value is 50. Moreover, the *Neofusicoccum* genus, consisting here of three species was isolated from eight plants, one of which revealed a CF% of 34.21. Interestingly, around 70% of the fungal species in *Artemisia thuscula* were isolated from only one plant each (Table 5). This suggests a host specificity which was also exhibited by the low and moderate values of Sorensen–Dice coefficient when the similarity of the endophytic assemblages was analyzed (see further Diversity indices for endophytic fungi in *Artemisia thuscula*).

34 endophytic fungal species were isolated from *Artemisia thuscula* (Table 6) and their frequency varied within a low range with two exceptions: *Alternaria alternata* (CF% = 18.71) and *Neofusicoccum* sp. 3 (CF% = 8.39).

Studies that are independent of fungal isolation and identification methods often revealed higher numbers of fungal species [49]. We purposely chose the culture method to further select endophytic fungi of high interest according to their biological activities. Our goal was to yield a large number of endophytes, and not to produce a complete species list of fungal endophytes in these *Artemisia* species. Nevertheless, the data obtained gave us an interesting fragment of knowledge about the communities of these microorganisms in their plant hosts.

In terms of endophytic fungal species CF%, the most isolated species was *Alternaria alternata* (CF = 18.71; eight of ten plants), as expected. It is a common saprobe found on various plants and other substrata worldwide [50,51] and has often been isolated as endophyte in previous studies [52,53,54,55]. Qian et al., 2014 [27] isolated endophytic fungi from *Artemisia argy* and found Pleosporales to be the most represented group, with three species of *Alternaria* present. It was found as the most predominant species in grasses [56] and various plants families, also [57]. Among dominant endophytic fungal species, we observed taxa like *Neofusicoccum* and *Preussia*. These genera of endophytic fungi were previously isolated from a wide range of host plants including *Artemisia* spp. [24,57,58,59,60].

Interestingly, it was observed a tendency on host specificity of most endophytic fungal species. In *Cirsium arvense* similarity in endophytic communities decreased with increasing intersite distance [61] while in *Holcus lanatus* the similarity between leaf and root myco-assemblages at the same location was lower than that observed in leaves at different locations [58]. Further, in leaf fungal communities the average number of species shared by any pair of location was 3.13 and in root assemblages was 1.73 out of an average of 12.2 species identified at each location [58]. 

Despite the dominant species, the rest of the endophytic fungal species reflect an unequal distribution of a certain endophytic species among plant individuals. This same issue was previously observed [58] but no definitive answer has been found. Some hypotheses were proposed like ubiquitous taxa with spatial dominance or selection of certain dependent on culture conditions [58]. In the case study on *Artemisia thuscula* (see Section 3.1.3) taxa such as *Preussia*, *Pestalotiopsis*, *Aplosporella*, *Chaetomium* and *Cladosporium* were isolated from only one nutrient medium out of the three media tested. Nevertheless, this is not a unique parameter, which should account for the determination of an endophytic taxa preference for a nutrient medium. One of the major variables which we consider is the rest of the community involved and their role in the interaction when the isolation performed. That is, which are the other taxa living in the same “space” (i.e., plated plant fragment) and we must consider if there are (i) fast-growing taxa versus slow growing taxa; (ii) nutrient deficiency or promoting medium for certain taxa, as well as (iii) the interaction between the taxa (i.e. antagonism).

#### 3.1.2. Diversity Indices for Endophytic Fungi in *Artemisia* *thuscula*

In the La Palma results of diversity, Margalef index revealed the highest value for species richness in San Bartolo (Margalef = 4.24) followed by El Granel (Margalef = 3.69). The Brillouin index agrees that the highest diversity is found in San Bartolo (Brillouin = 1.8) but Fisher’s alpha index shows a higher abundance of rare species in El Granel (Fisher’s alpha = 18.6) than in San Bartolo (Fisher’s alpha = 13.9). Diversity regarded as evenness was found to be similar in both localities (Simpson’s index: El Granel = 0.88 and San Bartolo = 0.87). In La Palma Island, San Bartolo locality was revealed as having the highest value for species richness and diversity. Yet, El Granel was shown as having a higher abundance of rare species (Fisher’s alpha: El Granel = 18.6 and San Bartolo = 13.9) and a higher value of evenness than San Bartolo (Simpson’s index: El Granel = 0.88 and San Bartolo = 0.87). In Tenerife, the locality San Andres showed by far the highest diversity in all previously mentioned terms and all the indices confirm it (Table 7).

**Figure 1:**
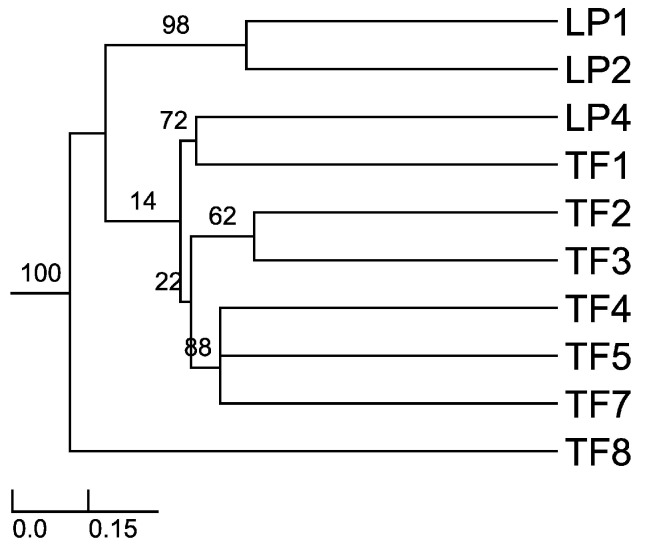
Sorensen’s similarity coefficient for the endophytic fungi isolated from *A. thuscula*: unweighted paired group method of arithmetic average (UPGMA) dendrogram plot. The results were obtained with 95% of confidence and bootstrap values calculated from 1000 iterations.

Sorensen–Dice index revealed that of 45 cases in the matrix, 27 of them were of zero similarity. Further, only one case was found to have 57% similarity (TF2 versus TF7) and one case with 50% similarity (TF1 versus TF4). The rest of the cases had values ranging between 11% and 40% similarity. These different similarity values may be due to distance among hosts, soil composition and/or climatic conditions. When the distance was plotted (UPGMA), the Sorensen–Dice coefficient clustered plants LP1 and LP2 with maximum bootstrap support (BPP = 100), although these plants had only 38% similarity in between. Nevertheless, this is to be considered a high value of similarity in the given matrix and one of the reasons for obtaining it might be the proximity of the collection places (approx. 5 km) between the host plants and similar altitudes and climate. Further clusters were formed like LP4 and TF1; TF2 and TF3; TF4, TF5 and TF7 (Figure 1). As we expected (from CF and CR values) TF8 is the most different host plant, the backbone of the dendrogram divides into this branch and the other branches which form various clusters of similarity. Also, cluster LP1 and LP2 is a sister cluster of the other clusters which were exhibited as more related in terms of similarity.

#### 3.1.3. Case Study: *Artemisia thuscula* of Tenerife, Endophytic Fungi Isolated from Two Types of Stems on Three Media: Colonization Frequency and Colonization Rate

In this study, we can observe throughout various individual plants from the same species (i.e., *Artemisia thuscula*) the relevance of nutrient media and the age of the stem as the selected organ to yield endophytic fungi. When averaged the colonization rates of the three nutrient media selected (PDA, LCA, and V8) do not show relevant differences (CR% = 33.93. 33.93 and 37.50, respectively). Neither do the differences of age in stems; stems with the age < 1 year have CR% = 30.95 and stems with age > 1 year have CR% = 36.90.

Differences may be observed (Table 8) when comparing different plants, as for instance TF3 and TF4 had the lowest colonization rates (CR% = 16.67) and no endophytic fungi was isolated from V8 or stems with age of more than 1 year for TF4 and TF3, respectively. In addition, there is no higher value than 58.33 of colonization rate, as observed in other individuals of *Artemisia*.

If colonization frequency data is segregated into plants sampled (Table 9), we observe that *Alternaria alternata* is the major colonizer in three out of seven plants, namely TF2, TF4, and TF5. Plants had different yields considering number of endophytic fungal species, ranging between three (TF3) and eight (TF7).

Among the major colonizers we observed *Neofusicoccum austral* and *Neofusicoccum parvum* in TF3 (CF% = 8.33), *Chaetomium* sp. 1 and *Phoma* sp. 1 in TF7 and *Phoma* with two different species in TF8 (CF% = 16.67; Table 8). Myrchiang et al., 2014 [28] investigated the endophytic fungi associated with *Artemisia nilagirica* and comparing the colonization of three organs (i.e., root, stem and leaf), the authors obtained the highest diversity in the roots (i.e., 14 species), less in stem (i.e., 10 species) and the smallest number in the leaves (i.e., 6 species). Similarly, in *Artemisia thuscula* Cosoveanu et al., 2012 [62] isolated 29 distinct morphotypes: 20 from roots, 7 from stem and 2 from leaves. In addition, Myrchiang et al., 2014 [28] observed that from all fungal endophytic species, only *Phoma eupyrena* was found to be a common occurrence in all plants sample, the other species having a certain preference for one or maximum two organs.

Comparing different plant individuals of the same species and observing the distribution of fungal endophytes provides insights to determine the occurrence of a certain species. For instance, in TF2 four fungal species were isolated only from one nutrient medium, namely *Biscogniauxia mediterranea* in PDA, *Alternaria* sp. on LCA, *Phoma* sp. on PDA and *Pestalotiopsis* sp. on LCA (Table 9). Furthermore, we may observe that the same species of *Phoma* sp. 1 was also isolated from TF8 on PDA, similar to *Pestalotiopsis* isolated from TF3 on LCA while *Biscogniauxia mediterranea* was isolated on V8 from TF8.

**Figure 2:**
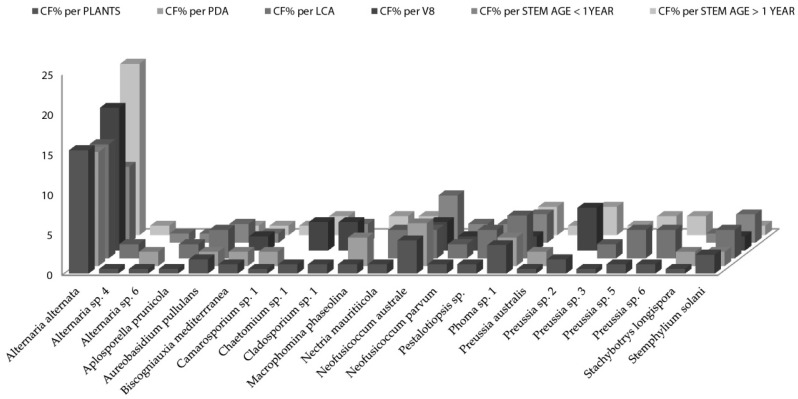
*Artemisia thuscula* colonization frequencies of endophytic fungi species per total number of plant individuals, per media (PDA, lignocellulose agar (LCA) and V8) and per stem age (>1 year and <1year). The *y*-axis data correspond to colonization frequency percentage.

When the distribution of endophytic fungi species is observed in terms of colonization frequency per total number of the studied plants (Figure 2), data showed several species like *Aplosporella prunicola*, *Camarosporium* sp., *Chaetomium* sp., *Cladosporium* sp, *Nectria mauritiicola* and others with certain “preference” for nutrient medium. It is well known that fungi have specific carbon and nitrogen requirements for sporulation [63,64,65]. However, the requirements for fungal growth are less stringent but not less important when isolation is pursued. Nutrient - rich media result in selective isolation for fast-growing fungi, overlooking slow growing species if present [66]. Osono and Takeda [29] stated that LCA due to its low glucose content suppresses the overgrowth of fast-growing species. 22 species of fungal endophytes were isolated from all *Artemisia thuscula* plants in this case study and 14 species (63%) were isolated only from one nutrient medium. Additionally, 12 fungal species were isolated from stems older than 1 year and seven were isolated from stems younger than one year. Seven fungal species are to be considered rare, as their colonization frequency value is the lowest one, throughout the data set (CF% = 0.60; Figure 2).

Further, Sorensen–Dice similarity coefficient reveals proximate values among the similarities of the endophytic communities isolated on the three tested media (Table 10). Yet, none of them overpassed 52% similarity (i.e., LCA versus V8). As for the stem ages, the index showed a value of 43% similarity. Evidence for tissue specificity was previously demonstrated for phloem and xylem tissue, where the value of endophytic similarity reached 36% in roots of *Sophora tonkinensis* [67]. This suggests the necessity to broad both culture media and diversity of tissues to obtain a higher richness of endophytic fungal taxa.

Among the singleton species that occur only in the *Artemisia thuscula* plant individuals selected for this case study (i.e., limited to Tenerife) we have: *Aplosporella prunicola*, *Camarosporium* sp. 1, *Macrophomina phaseolina*, *Chaetomium* sp. 1, *Nectria mauritiicola*, *Neofusicoccum australe*, *Pestalotiopsis* sp., *Phoma* sp. 1 and *Stachybotrys longispora*. Except *Phoma* sp., all endophytic fungal species previously mentioned were isolated from only one nutrient medium. In addition, except *Camarosporium* sp. (isolated also from *Artemisia thuscula* in Palma Island), all endophytic fungal species previously named were isolated only from *Artemisia thuscula* in Tenerife Island.

### 3.2. Phylogenetic Relations

54 endophytic fungal ITS sequences and the associated GenBank sequences were used for the phylogenetic analysis (Table 3; sequences of strains HLP16, HLP22, HLP28, HLP33, HLP48A, HLP4, HTF29, HTF33, HTF43, and HTF61 are not listed and are only available at request). The dataset consists of 603 characters after alignment, 43 characters are conserved, and 447 characters are parsimony informative, while 557 are variable characters. Bayesian Posterior Probabilities (BPPs) given below each node are shown on the upper branches.

**Figure 3:**
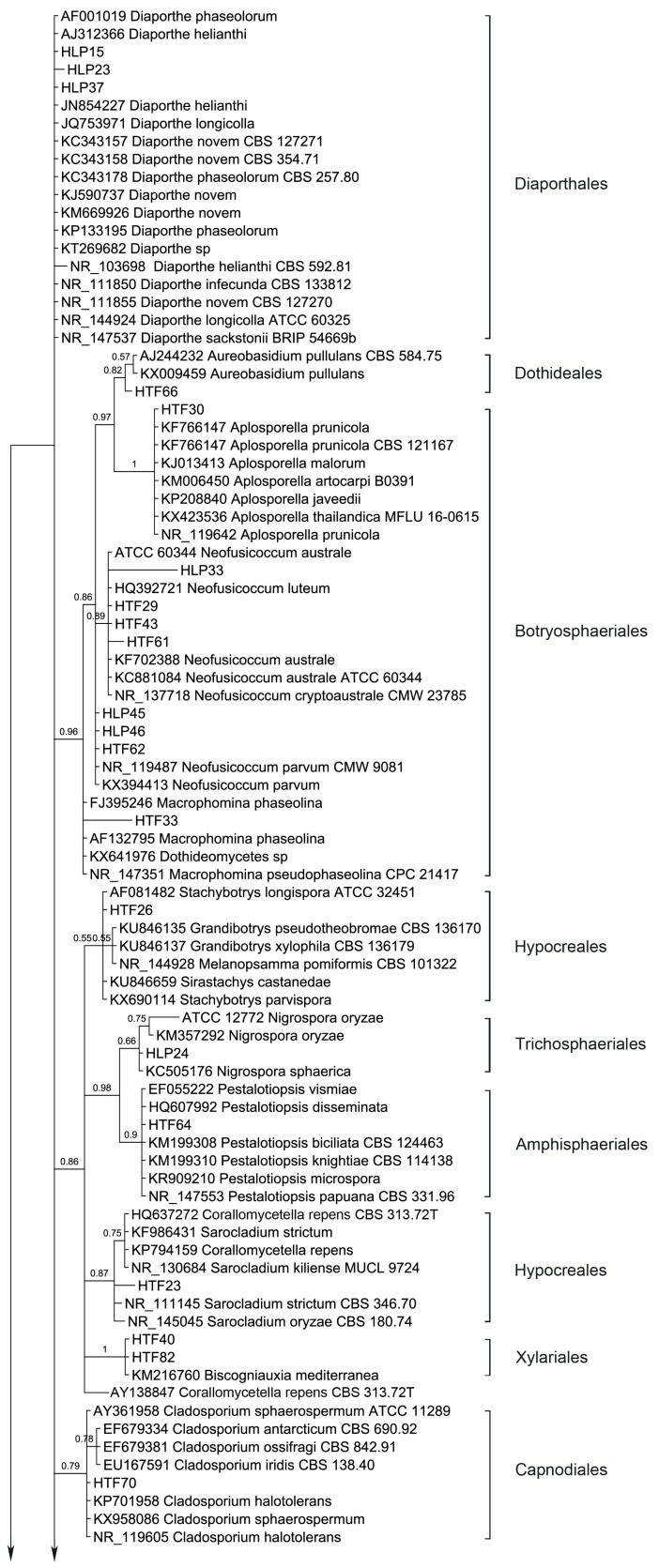

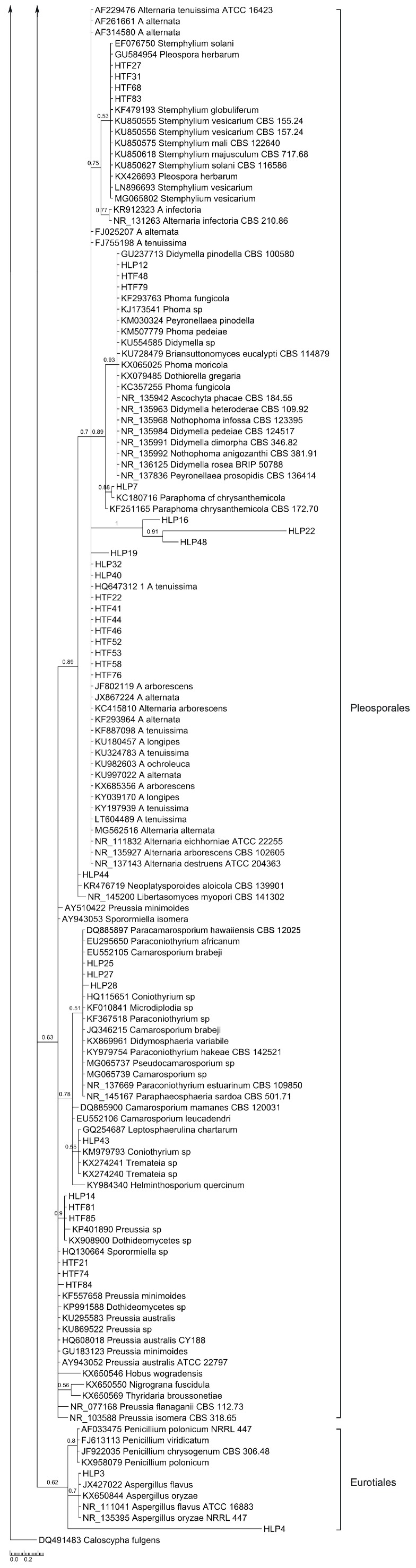
Bayesian phylogenetic tree based on ITS rDNA sequence variants of the endophytic fungi isolated from *A. thuscula* and their associated external GenBank hits. The tree was rooted with *Caloscypha fulgens* sequence as outgroup. The Bayesian clade-credibility values (posterior probabilities) are indicated at internodes (BPP). The scale bar represents the expected changes per site. Sequences coded with HLP/HTF were obtained from endophytic fungi, sequences coded with taxa names are associated external sequences and the ones coded with CBS/L/ATCC/NRRL/B/MFLU/CMW/MUCL/BRIP were obtained from type strains.

Ten orders (Diaporthales, Dothideales, Botryosphaeriales, Hypocreales, Trichosphaeriales, Amphisphaeriales, Xylariales, Capnodiales, Pleosporales and Eurotiales) are recognized (Figure 3). The phylogenetic tree divides the taxa in five main clades, leaving *Diaporthe* sequences unclustered. Clade 1 consists of Dothideales and Botryosphaeriales (BPP = 0.98), Clade 2 groups Hypocreales, Trichosphaeriales, Amphisphaeriales and Xylariales (BPP =0.88), Clade 3 and Clade 4 contain Capnodiales (BPP = 0.79) and Pleosporales (BPP = 0.63), respectively while Clade 5 accommodates Eurotiales (BPP = 0.62).

Interestingly, *Diaporthe* sequences are not clustered but several show different branch lengths. Yet, taxa *D. novem* and *D. phaseolorum* do not differentiate. Endophytic fungi were basically identified using morphology; therefore, HLP15 and HLP23 were considered *D. phaseolorum* and *D. novem*, respectively while structures of HLP37 did not allow an accurate species level identification. Apparently, the ITS region in *Diaporthe* is evolving at higher rates than TEF1 or MAT genes [68], therefore presenting a wider variation than advisable for species boundaries. Thus, a slowly evolving gene region should be used in order to establish species limits [69]. Nevertheless, ITS sequence data can be used for reliable identification of phylogenetic relationships as long as they are interpreted with care [69]. Several arrangements of genera draw the attention, like *Aureobasidium* (Dothideales) and *Aplosporella* (Botryosphaeriales) which are shown with an immediate common ancestor (BPP = 0.97). *Aplosporella* has over 300 species and appears to be heterogenous; therefore not all species are likely to belong in Botryosphaeriaceae [70]. The ascomycete genus *Aureobasidium* is a member of the family Aureobasidiaceae within the class of the Dothideomycetes [71]. Dothideomycetidae subclass was emended by Schoch et al., 2006 [72] and a new subclass was proposed, Pleosporomycetidae, with an additional order, the Botryosphariales.

*Penicillium* and *Aspergillus* sequences form two sister clades as expected (BPP = 0.60). Three species of *Neofusicoccum* are clustered with relevant support (BPP = 0.89) while *N. parvum* is drawn outside. Hypocreales taxa are split in two sister clusters along with *Stachybotrys*, *Grandibotrys*, *Melanopsamma* and *Sirastachys* in one sister clade although with no relevant support (BPP = 0.55) and *Nectria*, *Sarocladium* and *Corallomycetela* as another sister clade (BPP = 0.87). Also, internal clustering is revealed between several taxa of the mentioned genera. Trichosphaeriales and Amphisphaeriales are shown having a common recent ancestor (BPP = 0.98). Hypocreales is recognized as monophyletic [73]. The order Hypocreales incorporates Nectriaceae and Stachybotriaceae beyond other six families [74]. Maharachchikumbura et al., 2014 [75] found using a combined LSU, SSU, TEF and RPB2 sequences data that *Stachybotrys* and related taxa (Stachybotriaceae) form a sister cluster of *Nectria* and related taxa (Nectriaceae). The results obtained with the ITS region are in accordance with the combined inference obtained by Maharachchikumbura et al., [75]. The Nectriaceae group (BPP = 0.87) comprises *Nectria* (Nectriaceae), *Sarocladium* (Hypocreomycetidae) and *Corallomycetella*—shown to comprise two distinct clades in Nectriaceae [76]. The second cluster joints *Stachybotrys*, *Grandibotrys*, *Sirastachys*, *Stachybotrys* (Stachybotriaceae, Hypocreomycetidae) and *Melanopsamma* (Chaetosphaeriaceae, Sordariomycetidae). *Melanopsamma pomiformis* was recently excluded from the genus [77] and it was linked to the asexual morph *Stachybotrys albipes* [78]. Strains of Sordariomycetes clustered into six subclasses among which Diaporthomycetidae, Xylariomycetidae and Hypocreomycetidae [75]. Our Bayesian analysis resulted in a monophyletic clade (Clade 2) which accommodates Hypocreales (Hypocreomycetidae), Trichosphaeriales (Diaporthomycetidae), Amphisphaeriales (Xylariomycetidae) and Xylariales (Xylariomycetidae). Yet, Diaporthales taxa (*Diaporthe* spp.) were left outside this clade. A resulting parsimonious tree of multi-locus based (LSU, ITS, and *TEF1*) sequences shows that the genus *Diaporthe* has paraphyletic origins [79]. Xylariales and Amphisphaeriales were found as sister clusters in Xylariomycetidae sharing a common ancestor [80]. Yet, the clade which accommodates Xylariomycetidae is a sister clade of Diaporthomycetidae (Diaporthales) and Hypocreomycetidae (Hypocreales).

*Cladosporium* sequences are clustered (BPP = 0.79) and different branch lengths between species are revealed, grouping *C. ossifragi*, *C. antarcticum* and *C. iridis* (BPP = 0.78). Conversely, *Aplosporella* sequences do not differentiate in between, showing all species with same branch lengths.

*Stemphylium* sequences are grouped but support does not avail this grouping (BPP = 0.53). *Phoma*-like sequences are clustered as expected (BPP = 0.93) showing higher differences between *Phoma*, *Didymella*, *Dothiorella* and *Notophoma* on one side (BPP = 0.93) and *Paraphoma chrysantemicola* on the other side (BPP = 0.88). It is curious that several sequences of endophytes are grouped in a sister clade of *Alternaria* clade, *Phoma*-like clade and *Stemphylium* clade with high probability (BPP = 1), indicating different branch lengths. *Alternaria* sequences are not grouped in a single cluster but different branch lengths are drawn among the species. Similarly, *Preussia* and *Sporormiella* taxa are spread. *Coniothyrium*-like sequences are clustered, but support has an average value, BPP = 0.78. Coniothyriaceae and Camarosporiaceae grouping as well as the *Coniothyrium*-like sequences cluster and its sister cluster of Pleosporaceae is supported by the findings of Wijayawardene et al., 2014 [81]. Mainly the sequences obtained from the endophytic strains are grouped with the external sequences as expected (i.e., morphological identification) but several are left unclustered. For instance, inside the group of Pleosporales three endophytic sequences (HLP16, HLP22 and HLP48A) appear as more related, forming a strong-supported cluster (BPP = 1). This apparently new lineage should be confirmed with another phylogenetic study based on large subunit and small subunit nuclear rDNA regions, where only Pleosporales taxa would be included. In the present study none of the methods used like the morphology (absence of the sporulating structures), BLAST alignment (values of similarity with GenBank provided sequences did not exceed 86%, 88% and 84% for HLP16, HLP22, and HLP48A, respectively) and the ITS inference, could provide their proper identification or genetic stronger alliances inside Pleosporales.

## 4. Conclusions

The present study suggests culturable endophytic species have specificity for a plant host and “preference” for nutrient medium. Therefore, this study indicates the apparent necessity of using different culture media so as to obtain a higher diversity of species.

## Figures and Tables

**Table 1:** Details of collected *Artemisia* species plants.

Plant Species	Plant Code	Collection Place	Country/Island	Herbarium Type Details	GPS UTM Latitude	GPS UTM Longitude
*A. thuscula*	LP1	El Granel	La Palma	TFC. No. 52658	28°45′47.43″ N	17°45′7.47″ W
*A. thuscula*	LP2	San Bartolo	La Palma	TFC. No. 52659	28°46′1.08″ N	17°45′26.07″ W
*A. thuscula*	LP4	Tigalate	La Palma	TFC. No. 52661	28°32′35.45″ N	17°48′41.29″ W
*A. thuscula*	TF8	El Palmar	Tenerife	TFC. No. 52669	28°20′35.18″ N	16°51′26.57″ W
*A. thuscula*	TF7	Granadilla	Tenerife	TFC. No. 52668	28°06′54.19″ N	16°34′51.14″ W
*A. thuscula*	TF4	Caletillas	Tenerife	TFC. No. 52665	28°23′2.03″ N	16°21′54.71″ W
*A. thuscula*	TF1	Mesa Mota	Tenerife	TFC. No. 52662	28°30′38.75″ N	16°19′20.55″ W
*A. thuscula*	TF2	Mesa Mota	Tenerife	TFC. No. 52663	28°30′38.75″ N	16°19′20.55″ W
*A. thuscula*	TF5	San Andres	Tenerife	TFC. No. 52666	28°30′51.01″ N	16°11′41.94″ W
*A. thuscula*	TF3	Taborno	Tenerife	TFC. No. 52664	28°33′18.36″ N	16°15′53.10″ W

**Table 2:** *Artemisia* fungal endophytic strains: codes, identities as per morphology, 28S rDNA LSU sequences and their most similar hits from Genbank with accession numbers and values.

EF Code	Assigned Species/Species Complex	GenBank Identified Seq.	Max Score	Total Score	E Value	Max Identity (%)	Accession
							No.
HLP1	*A. alternata*	*A. alternata*	1107	1107	0	99	KX609781.1
HLP10	*A. alternata*	*A. alternata*	1093	1093	0	99	KX609781.1
HLP31	*Fungus* sp. 1	Uncultured fungus clone	135	724	2.00 × 10^−27^	100	KP843503.1
HLP5	*A. alternata*	*A. alternata*	1052	1052	0	99	KF751621.1
HLP6	*Curvularia lunata*	*Cochliobolus lunatus*	1026	1026	0	99	KC616350.1
HLP8	*Neofusicoccum* sp. 1	*N. cryptoaustrale*	511	511	5.00 × 10^−141^	92	KX464415.1
HLP9	*Preussia* sp. 1	*P. mimoides*	1000	1000	0	97	KF557659.1
HTF25	*Alternaria* sp. 6	*A. brassicicola*	289	289	5.00 × 10^−74^	77	AF397222.1
HTF37	*A. alternata*	*A. alternata*	982	982	0	96	KX609781.1
HTF42	*Neofusicoccum australe*	*N. australe*	1036	1036	0	97	KF766367.1
HTF49	*Neofusicoccum australe*	*N. australe*	1058	1058	0	98	HM176550.1
HTF50	*A. alternata*	*A. alternata*	971	971	0	98	KF543048.1
HTF67	*Chaetomium* sp. 1	*C. coarctatum*	846	846	0	99	KX976729.1
HTF75	*Neofusicoccum australe*	*N. australe*	934	934	0	98	HM176550.1
HTF78	*A. alternata*	*A. alternata*	1051	1098	0	99	FJ839651.1
HTF80	*Camarosporium* sp. 1	*Camarosporium* sp.	1024	1024	0	97	KF733369.1

**Table 3:** Endophytic fungi isolated from *A. thuscula* and used for the phylogenetic analysis: codes, identity and accession numbers of the ITS sequences.

Strain Code	Identity	Accession No.
HLP12	*Phoma* sp.	MG025848
HLP14	*Preussia* sp.	MG025849
HLP15	*Diaporthe phaseolorum*	MG025850
HLP19	*Alternaria alternata*	MG025851
HLP23	*Diaporthe novem*	MG025852
HLP24	*Nigrospora oryzae*	MG025853
HLP25	*Camarosporium brabeji*	MG025854
HLP27	*Coniothyrium* sp.	MG025855
HLP3	*Aspergillus flavus*	MG025856
HLP32	*Alternaria alternata*	MG025857
HLP37	*Diaporthe* sp.	MG025858
HLP40	*Alternaria alternata*	MG025859
HLP43	*Tremateia* sp.	MG025860
HLP44	*Neoplatysporoides aloicola*	MG025861
HLP45	*Neofusicoccum parvum*	MG025862
HLP46	*Neofusicoccum parvum*	MG025863
HLP7	*Paraphoma chrysanthemicola*	MG025864
HTF23	*Nectria mauritiicola*	MG025865
HTF26	*Stachybotrys longispora*	MG025866
HTF27	*Stemphylium solani*	MG025867
HTF30	*Aplosporella prunicola*	MG025868
HTF31	*Stemphylium solani*	MG025869
HTF40	*Biscogniauxia mediterranea*	MG025870
HTF41	*Alternaria alternata*	MG025871
HTF44	*Alternaria alternata*	MG025872
HTF46	*Alternaria alternata*	MG025873
HTF48	*Phoma* sp.	MG025874
HTF52	*Alternaria alternata*	MG025875
HTF53	*Alternaria alternata*	MG025876
HTF62	*Neofusicoccum parvum*	MG025877
HTF64	*Pestalotiopsis* sp.	MG025878
HTF66	*Aureobasidium pullulans*	MG025879
HTF68	*Stemphylium solani*	MG025880
HTF70	*Cladosporium* sp.	MG025881
HTF74	*Preussia australis*	MG025882
HTF76	*Alternaria alternata*	MG025883
HTF79	*Phoma* sp.	MG025884
HTF81	*Preussia* sp.	MG025885
HTF82	*Biscogniauxia mediterranea*	MG025886
HTF83	*Stemphylium solani*	MG025887
HTF84	*Preussia* sp.	MG025888
HTF85	*Preussia* sp.	MG025889

**Table 4:** Colonization rate (CR) of fungal endophytes in *Artemisia thuscula* collected in Canary Islands.

Collection Place	Region	Plant Number	Locality Code	CR%
El Granel	La Palma	*LP1	EG	48.28
El Palmar—Teno	Tenerife	**TF8	EP	25.00
Granadilla	Tenerife	TF7	GR	25.00
Igueste Caletillas	Tenerife	TF4	IC	50.00
Mesa Mota	Tenerife	TF1	MM	62.50
Mesa Mota	Tenerife	TF2	MM	62.50
San Andrés	Tenerife	TF5	SA	62.50
San Bartolo	La Palma	LP2	SB	92.11
Taborno	Tenerife	TF3	TA	25.00
Tigalate	La Palma	LP4	TIG	55.00
AVG				*50.78*
SD				*16.13*

*LP = La Palma; **TF = Tenerife; AVG = average; SD = standard deviation.

**Table 5:** Colonization frequency on potato dextrose agar (PDA) medium of fungal endophytic species in *Artemisia thuscula* plants.

Plant Code	Locality Code	EF Species	CF%	Plant Code	Locality Code	EF Species	CF%
LP1	EG	*Alternaria alternata*	24.14	LP2	SB	*Preussia* sp. 3	2.63
LP1	EG	*Alternaria* sp. 5	13.79	LP2	SB	*Tremateia* sp. 1	2.63
LP1	EG	*Aspergillus flavus*	3.45	LP4	TIG	*Alternaria alternata*	15.00
LP1	EG	*Aspergillus flavus*	6.90	LP4	TIG	*Curvularia lunata*	5.00
LP1	EG	*Diaporthe novem*	3.45	LP4	TIG	*Neofusicoccum* sp. 1	5.00
LP1	EG	*Fungus* sp. 1	3.45	LP4	TIG	*Paraphoma* cf. *chrysantemicola*	5.00
LP1	EG	*Neofusicoccum parvum*	3.45	LP4	TIG	*Preussia* sp. 1	25.00
LP1	EG	*Nigrospora oryzae*	3.45	TF1	MM	*Alternaria alternata*	25.00
LP1	EG	*Penicillium viridicatum*	3.45	TF1	MM	*Thielavia* sp. 1	8.33
LP1	EG	*Phoma* sp. 3	3.45	TF2	MM	*Alternaria alternata*	50.00
LP1	EG	*Pleosporales* sp. 2	6.90	TF2	MM	*Biscogniauxia mediterrranea*	12.50
LP1	EG	*Preussia* sp. 3	3.45	TF2	MM	*Neofusicoccum australe*	12.50
LP2	SB	*Alternaria alternata*	10.53	TF2	MM	*Phoma* sp. 1	12.50
LP2	SB	*Alternaria* sp. 5	2.63	TF3	TA	*Neofusicoccum australe*	25.00
LP2	SB	*Camarosporium bradgi*	2.63	TF4	IC	*Alternaria alternata*	37.50
LP2	SB	*Coniothyrium* sp. 1	2.63	TF4	IC	*Aureobasidium pullulans*	12.50
LP2	SB	*Diaporthe phaseolorum*	7.89	TF5	SA	*Alternaria alternata*	12.50
LP2	SB	*Diaporthe* sp. 1	5.26	TF5	SA	*Alternaria* sp. 6	12.50
LP2	SB	*Dothideomycetes* sp. 1	2.63	TF5	SA	*Macrophomina phaseolina*	25.00
LP2	SB	*Fungus* sp. 1	2.63	TF5	SA	*Stachybotrys longispora*	12.50
LP2	SB	*Neofusicoccum parvum*	13.16	TF7	GR	*Alternaria alternata*	25.00
LP2	SB	*Neofusicoccum* sp. 3	34.21	TF7	GR	*Neofusicoccum australe*	25.00
LP2	SB	*Neoplatysporoides aloicola*	5.26	TF7	GR	*Stemphylium solani*	25.00
LP2	SB	*Nigrospora* sp. *2*	2.63	TF8	EP	*Camarosporium* sp. 1	12.50
LP2	SB	*Pleosporales* sp. *3*	2.63	TF8	EP	*Phoma* sp. 1	12.50

EG = El Granel; SB = San Bartolo; TIG = Tigalate; MM = Mesa Mota; TA = Taborno; IC = Igueste Caletillas; SA = San Andres; GR = Granadilla; EP = El Palmar.

**Table 6:** Colonization frequency of fungal endophytic species in *Artemisia thuscula* (overall CF%/plant species).

EF Species	CF%	EF Species	CF%
*Alternaria alternata*	18.71	*Neofusicoccum parvum*	3.87
*Alternaria* sp. 5	3.23	*Neofusicoccum* sp. 1	0.65
*Alternaria* sp. 6	0.65	*Neofusicoccum* sp. 3	8.39
*Aspergillus flavus*	1.94	*Neoplatysporoides aloicola*	1.29
*Aureobasidium pullulans*	0.65	*Nigrospora oryzae*	0.65
*Biscogniauxia mediterrranea*	0.65	*Nigrospora* sp. 2	0.65
*Camarosporium bradgi*	0.65	*Paraphoma chrysantemicola*	0.65
*Camarosporium* sp. 1	0.65	*Penicillium viridicatum*	0.65
*Coniothyrium* sp. 1	0.65	*Phoma* sp. 1	1.29
*Curvularia lunata*	0.65	*Phoma* sp. 3	0.65
*Diaporthe novem*	0.65	*Pleosporales* sp. 2	1.29
*Diaporthe phaseolorum*	1.94	*Pleosporales* sp. 3	0.65
*Diaporthe* sp. 1	1.29	*Preussia* sp. 1	3.23
*Dothideomycetes* sp. 1	0.65	*Preussia* sp. 3	1.29
Fungus sp. 1	1.29	*Stachybotrys longispora*	0.65
*Macrophomina phaseolina*	1.29	*Stemphylium solani*	0.65
*Neofusicoccum australe*	2.58	*Tremateia* sp. 1	1.94

**Table 7:** Diversity indices of fungal endophytic species per plant individual/locality.

Locality Code	Taxa No.	Strains No.	Simpson 1-D	Brillouin	Margalef	Fisher Alpha
LP1/EG	11	15	0.89	1.65	3.69	18.60
LP2/SB	15	27	0.87	1.85	4.25	13.90
LP4/TIG	5	5	0.80	0.96	2.49	0.00
TF1/MM	5	10	0.60	0.85	1.74	3.98
TF3/TA	1	2	0.00	0.00	0.00	0.80
TF4/IC	2	3	0.44	0.37	0.91	2.62
TF5/SA	4	5	0.72	0.82	1.86	9.28
TF7/GR	3	3	0.67	0.60	1.82	0.00
TF8/EP	2	2	0.50	0.35	1.44	0.00

LP1/EG = El Granel; LP2/SB = San Bartolo; LP4/TIG = Tigalate; TF1/MM = Mesa Mota; TF3/TA = Taborno; TF4/IC = Igueste Caletillas; TF5/SA = San Andres; TF7/GR = Granadilla; 14/EP = El Palmar.

**Table 8:** *Artemisia thuscula* colonization rate (CR%) of endophytic fungi per plant. per medium and per stem age.

Plant Code	CR%/Plant	CR%/PDA	CR%/LCA	CR%/V8	CR%/Stem	
					Age < 1 Year	Age > 1 Year
TF3	16.67	25	12.5	12.5	33.33	0
TF4	16.67	37.5	12.5	0	8.33	25
TF5	58.33	62.5	75	37.5	58.33	41.67
TF7	45.83	25	50	62.5	50	41.67
TF8	37.5	25	37.5	50	8.33	66.67
AVG	35.12	33.93	33.93	37.50	30.95	36.90

**Table 9:** *Artemisia thuscula* colonization frequency (CF%) of endophytic fungi species per plant, per medium and per stem age.

EF Species	Plant	CF%/Plant	CF%/Medium			CF%/Stem Age	
			PDA	LCA	V8	≤1 year old	>1 year old
*Alternaria alternata*	TF2	50	50	37.5	62.5	41.67	58.33
*Alternaria* sp. 4		4.17		12.5			8.33
*Biscogniauxia mediterrranea*		4.17	12.5			8.33	
*Neofusicoccum australe*		16.67	12.5	12.5	12.5	16.67	8.33
*Pestalotiopsis* sp.		4.17		12.5		8.33	
*Phoma* sp. 1		4.17	12.5				8.33
*Neofusicoccum australe*	TF3	8.33	25			16.67	
*Neofusicoccum parvum*		8.33		12.5	12.5	16.67	
*Pestalotiopsis* sp.		4.17		12.5		8.33	
*Alternaria alternata*	TF4	12.5	37.5			16.67	8.33
*Aureobasidium pullulans*		4.17	12.5			8.33	
*Nectria mauritiicola*		8.33		25			16.67
*Alternaria alternata*	TF5	37.5	12.5	50	50	8.33	66.67
*Alternaria* sp. 6		4.17	12.5			8.33	
*Aplosporella prunicola*		4.17		12.5		8.33	
*Aureobasidium pullulans*		4.17		12.5			8.33
*Macrophomina phaseolina*		8.33	25				16.67
*Neofusicoccum australe*		4.17		12.5		8.33	
*Stachybotrys longispora*		4.17	12.5			8.33	
*Stemphylium solani*		8.33		12.5	12.5	16.67	
*Alternaria alternata*	TF7	8.33	8.33	12.5	12.5		16.67
*Aureobasidium pullulans*		12.5		12.5		8.33	
*Chaetomium* sp. 1		16.67			25		16.67
*Cladosporium* sp. 1		12.5			25	16.67	
*Neofusicoccum australe*		8.33			12.5		8.33
*Phoma* sp. 1		16.67		50		33.33	
*Preussia australis*		8.33	12.5				8.33
*Stemphylium solani*		4.17	12.5			8.33	
*Biscogniauxia mediterrranea*	TF8	8.33			12.5		8.33
*Camarosporium* sp. 1		8.33	12.5				8.33
*Phoma* sp. 1		16.67	12.5			8.33	
*Preussia* sp. 2		16.67			37.5		25
*Preussia* sp. 3		12.5		12.5			8.33
*Preussia* sp. 5		12.5		25			16.67
*Stemphylium solani*		4.17		12.5			8.33

**Table 10:** *A. thuscula* fungal endophytes isolated on different nutrient media and stem age: Sorensen–Dice coefficient of similarity.

	LCA	V8	≤1 Year	>1 Year
PDA	0.42	0.48	0.64	0.71
LCA		0.52	0.67	0.67
V8			0.67	0.59
≤1 year				0.43
